# Factors Associated with Late Local Radiation Toxicity after Post-Operative Breast Irradiation

**DOI:** 10.1155/2022/6745954

**Published:** 2022-04-16

**Authors:** M. C. T. Batenburg, M. Bartels, W. Maarse, A. Witkamp, H. M. Verkooijen, H. J. G. D. van den Bongard

**Affiliations:** ^1^Department of Radiation Oncology, University Medical Center (UMC) Utrecht, Heidelberglaan 100, 3584 CX Utrecht, Netherlands; ^2^Department of Plastic, Reconstructive and Hand Surgery, University Medical Center Utrecht, Utrecht, Netherlands; ^3^Department of Surgery, University Medical Center Utrecht, Utrecht, Netherlands; ^4^Imaging Division, University Medical Center Utrecht, Utrecht, Netherlands; ^5^Department of Radiation Oncology, Amsterdam University Medical Centers, Amsterdam, Netherlands

## Abstract

**Purpose:**

To assess determinants associated with late local radiation toxicity in patients treated for breast cancer.

**Methods:**

A systematic review was performed. All studies reporting ≥2 variables associated with late local radiation toxicity after treatment with postoperative whole breast irradiation were included. Cohort studies, randomized controlled trials, and cross-sectional studies were eligible designs. Study characteristics and definitions of determinants and outcome measures were extracted. If possible, the measure of association was extracted.

**Results:**

Twenty-one studies were included in this review. Six out of seven studies focused on the association between radiotherapy (boost) dose or irradiated breast volume and late radiation toxicity found significant results. Tumor bed boost was associated with late radiation toxicity, fibrosis, and/or edema in six out of twelve studies. Lower age was associated with late breast toxicity in one study, while in another study, higher age was significantly associated with breast fibrosis. Also, no association between age and late radiation toxicity was found in eight out of twelve studies. Similar inconsistent results were found in the association between late radiation toxicity and other patient-related factors (i.e., breast size, diabetes mellitus) and surgical and systemic treatment-related factors (i.e., complications after surgery, chemotherapy, and time between surgery and radiotherapy).

**Conclusion:**

In modern 3D radiotherapy, radiotherapy (boost) dose and volume are—like in 2D radiotherapy—associated with late local radiation toxicity, such as breast fibrosis and edema. Treatment de-escalation, for example, partial breast irradiation in selected patients might be important to decrease late local toxicity without compromising locoregional control and survival.

## 1. Introduction

Due to early detection and improved treatments, a 5-year overall survival of women diagnosed with breast cancer in the Netherlands approaches 90% [1]. The large and growing number of breast cancer survivors calls for improved understanding of late effects of breast cancer treatment [2, 3]. For example, patients treated with radiotherapy might develop late radiation toxicity [4]. Late local radiation toxicity is characterized by breast or chest wall pain, breast fibrosis, impaired arm movement, breast or arm edema, or disappointing cosmetic results from at least 3 months after radiotherapy [4]. Previous systematic reviews focused on the association between impaired arm movement and late radiation toxicity, acute toxicity after late radiation toxicity, or the association between one determinant (e.g., breast size), and late radiation toxicity [5, 6]. However, no previous overview of the literature on the association of all determinants and breast/chest wall pain, fibrosis, and edema or overall late radiation toxicity were performed.

From studies investigating 2D radiotherapy, we know that higher radiotherapy dose and tumor bed boost are associated with more late radiation toxicity [7, 8]. In the 3D era of radiation therapy, various studies investigated the incidence of late toxicity within these new techniques to assess safety and long-term side effects. Simultaneously, new radiotherapy techniques and treatment de-escalation have been developed, such as implementation of intensity modulated radiotherapy-techniques, intraoperative radiotherapy, concurrent instead of sequential boost techniques, and hypofractionation [9–15].

In order to evaluate the toxicity profiles of these radiotherapy innovations, it is important to take prognostic factors that influence late toxicity into account. Previously, studies evaluated the association between one determinant and late radiation toxicity. For example, an increased radiotherapy dose or endocrine therapy is associated with a higher risk on breast fibrosis [8]. However, no overview of all clinical or radiotherapy-related factors associated with late radiation toxicity in the current 3D radiotherapy is known. Therefore, a systematic literature review was performed. The aim of this systematic review was to assess which factors (determinants) were associated with late radiation toxicity (outcome) of the breast in breast cancer patients (domain).

## 2. Methods

### 2.1. Search Strategy

A systematic literature search was performed in Pubmed and Embase on March 5, 2022. The following search terms, medical subject headings terms (MeSH), and their respective synonyms were combined: breast cancer AND late toxicity AND radiotherapy. Search terms were restricted to title and abstract. A complete search string is attached in Supplementary Material A. The preferred reporting items for systematic reviews and meta-analysis (PRISMA) guidelines were followed [16]. Both title and abstracts screening on eligibility criteria and full-text evaluation was performed independently by two authors. Disagreement on eligibility was solved by discussion and consensus.

### 2.2. Eligibility Criteria

Studies conducting research on predictive variables associated with late local radiation toxicity in irradiated breast cancer patients were eligible for inclusion. Predictive factors associated with late radiation toxicity of the skin might be correlated (e.g., chemotherapy and hormonal therapy). Therefore, only studies comparing multiple (i.e., ≥2) potential predictive variables were included. As late radiation toxicity is characterized by various symptoms, potentially the definition of late radiation toxicity differs per study. Subsequently, also studies evaluating the association between predictive variables and breast fibrosis, breast- or chest-wall pain, impaired arm movement, or breast/arm edema after radiotherapy were included. All cohort studies, including cohort studies in a trial population, randomized controlled trials (RCT), and cross-sectional studies were eligible designs. Case reports were excluded, as well as studies performed prior to 2005, since 3D-radiotherapy treatment performed nowadays is associated with different toxicity profiles than 2D-radiotherapy performed prior to 2005 [17]. Consequently, studies reporting 2D, but published after 2005 radiotherapy were also excluded.

Since external beam radiotherapy volumes and dosimetry in organs at risk differ from those in brachytherapy, cobalt radiotherapy, intraoperative radiotherapy, and proton radiotherapy, studies using these radiotherapy techniques were excluded. As irradiated breast volume is different in partial breast irradiation, resulting in less skin toxicity, studies conducting research on breast cancer patients treated with partial breast irradiation were excluded [18, 19]. This review focuses on local toxicity after postoperative irradiation in breast cancer patients. In this review, late local toxicity was defined as breast or chest wall pain, breast fibrosis, impaired arm movement, breast or arm edema, or disappointing cosmetic results from at least 3 months after radiotherapy [4]. Consequently, studies investigating cardiotoxicity, lung toxicity, and plexopathy caused by radiotherapy were excluded. The aim of this review was to assess the association between patient- or treatment-related factors and late local radiation toxicity. Therefore, studies conducting research on the association between genetic factors and late radiation toxicity were excluded. In addition, studies with a follow-up <12 months after treatment were excluded. Studies written in other languages than Dutch and English were excluded.

### 2.3. Critical Appraisal

The risk of bias of the included studies was assessed using the QUIPS risk of bias tool for prognostic studies [20]. In accordance with the Quality in Prognostic Studies (QUIPS) tool, risk of bias for all included studies was evaluated on six domains: “study participation,” “study attrition,” “prognostic factor measurement,” “outcome measurement,” “study confounding,” and “statistical analysis and reporting.” Each domain was rated “low,” “medium,” or “high” in accordance with proposed guidelines of Hayden et al. [20].

### 2.4. Data Extraction and Data Analysis

Characteristics extracted from the included studies were publication year, number of included patients, median age of participants, gender, tool used to determine toxicity, median follow-up duration, and study design. Also, radiotherapy planning technique, dose fractionation scheme, and total radiotherapy dose were extracted from all studies. The definition of all reported risk factors for each study was extracted. If possible, the measurement of association and strength of association for each variable was extracted. In case both univariable and multivariable analysis were performed, the results of the multivariable model were extracted as multivariable analysis was considered more reliable. When no measure of association was reported (e.g., only a *p*-value was provided), the strength of association was shown as not reported.

## 3. Results

**Figure 1:**
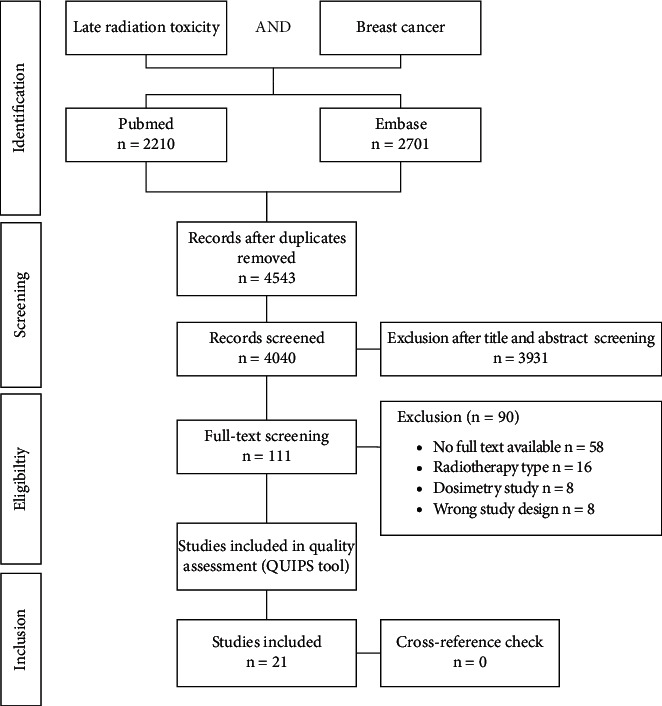
Flowchart of selected studies to evaluate which determinants were associated with late radiation toxicity in breast cancer patients.

The search strategy resulted in 4543 records. After exclusion of irrelevant records and records not meeting the in- and exclusion criteria, 21 studies were included in this review (Figure 1) [21–41].

**Figure 2:**
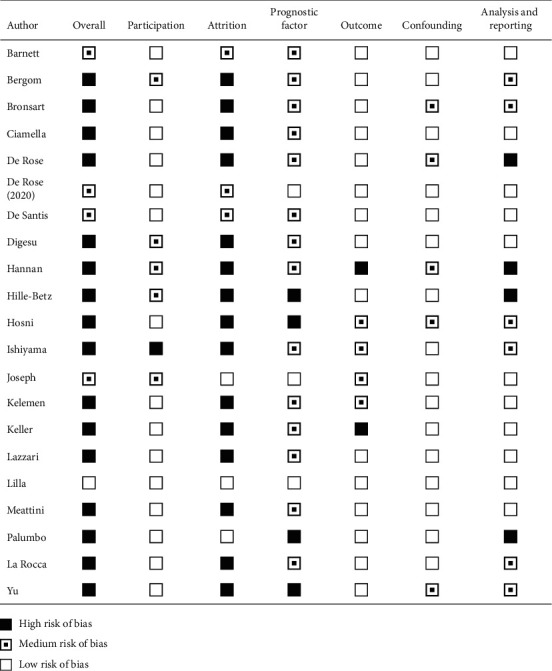
Risk of bias assessment for included studies using the quality in prognostic Studies (QUIPS) tool.

In accordance with the QUIPS tool, a high risk of bias was assigned to most studies due to high risk of bias in at least one of the subdomains of the QUIPS tool (Figure 2). High risk of bias was mostly caused by lack of description of missing data or lack of attempts to collect data from missing cases. Risk of bias assessment did not lead to exclusion of the studies for this review.

The included studies were published between 2007 and 2020. Follow-up time varied from 1–148.8 months. The majority (*n* = 17) of the studies had a median follow-up of >24 months (Table 1). In total, 8572 patients were included in all studies (range 67–1014 patients per study) and median age ranged from 49 to 74 years. In most studies, toxicity was assessed by a physician with Common Terminology Criteria for Adverse Events (CTCAE) [42] (*n* = 6), Radiation Therapy Oncology Group (RTOG) criteria [43] (*n* = 12), or Late Effects Normal Tissue-Subjective Objective Management Analytic (LENT-SOMA) [44] (*n* = 3). Most studies had a prospective design (*n* = 13/21, 62%). All patients were treated with postoperative whole breast irradiation (Table 2). In all the studies, patients were treated with 3D conformal radiotherapy, forward planned IMRT (*n* = 16) or inverse planned IMRT/VMAT (*n* = 5). Factors associated with late radiation toxicity were categorized into patient characteristics, factors related to radiotherapy, factors related to surgical or systemic treatment, or other (Table 3).

### 3.1. Association between Radiotherapy and Late Local Radiation Toxicity

Seven studies evaluated the association between increased radiotherapy volume or dose and late radiation toxicity [28, 30–32, 35, 37, 41] (Table 3). Six out of these seven studies found a significant association and the association measurement was quantified in four studies (Table 4). An association was seen between general radiation toxicity and breast volumes receiving >107% of the prescribed radiotherapy dose (OR 6.27 95%CI 1.34–29.37) [35]. Also, breast volumes receiving >110% of prescribed dose was correlated with higher late toxicity rates (R 0.402) [28]. In addition, they found that a planned target volume (PTV) > 1300 cc was highly correlated with general radiation toxicity in another study (R 0.955). In addition, an association between increased PTV volume and grade 2 fibrosis was shown (HR 1.14 95%CI 1.01–1.28) [37]. Another study defined radiation toxicity as either edema, erythema, or telangiectasia. They found a significant association between late radiation toxicity and an increase in clinical target volume (CTV) (respectively <500 vs. 500–900 vs. >900 OR 1.9 (95%CI not reported) and 3.0 (95%CI 2.0–4.5)) [41].

Several studies found a significant association between higher radiotherapy dose and breast fibrosis (Table 5). A radiotherapy dose of 50 Gy in 25 fractions resulted in a 12.5 times higher odds ratio than a dose of 30 Gy in 5 fractions (95%CI 2.73–57.13) [21]. However, three other studies found no significant association between radiotherapy dose or irradiated volume and fibrosis (Supplementary table B) [24, 36, 38]. Two studies found a significant association between (breast) pain and administration of additional radiotherapy boost on the tumor bed (respectively, OR 1.38 (95%CI 1.04–1.83) and 3.30 (95%CI 1.26–8.66)) (Table 4) [22, 39]. In addition, the administration of an additional radiotherapy boost on the tumor bed was significantly associated with higher breast fibrosis scores (OR 1.70 95%CI 1.16–2.48) (Table 5) [26]. Also, one study found that an increase in boost volume was associated with more fibrosis (OR 1.07 95%CI 1.00–1.14) [40]. Nevertheless, this association was not seen in three other studies (Supplementary Table B) [33, 36, 39].

Administration of a sequential boost to the tumor bed was associated with higher edema scores in the studies by Barnett et al., La Rocca et al., and Meattini et al. (respectively, OR 1.71 95%CI 1.20–2.43, 1.70 95%CI 1.08–2.67, and 9.02 (95%CI 1.21–67.45)) (Table 6) [22, 24, 26]. In addition, a significant association between higher boost volume and edema was seen in one study (OR 1.21 95%CI 1.09–1.33) [40]. One study showed significantly more edema when the boost dose was >16 Gy vs. <16 Gy (OR 1.9 95%CI 1.2–3.0) [41]. There were two studies that found no significant association between boost administration or boost dose and edema [24, 39].

### 3.2. Association between Surgical Treatment or Systemic Treatment and Late Radiation Toxicity

Two studies found an association between the occurrence of surgical complications and late radiation toxicity (Table 3) [22, 29]. Barnett et al. found a significant association between postoperative infection and late oversensitivity of the breast after radiotherapy and (OR 1.78 95%CI 1.27–2.49) (Table 7) [22]. Huang et al. found a significant association between postoperative complications and general radiation toxicity (Table 4) [29]. However, Ciamella et al. found no association between surgical complications and late radiation toxicity (Supplementary Table C) [35].

An association between axillary lymph node dissection and both arm and breast edema was seen in two studies [9, 12]. Chemotherapy was associated with increased edema scores in one study (OR 5.64 95%CI 1.18–26.98) [39]. Also, one study reported an increased OR for administration of chemotherapy and endocrine therapy of 2.3 (95%CI 1.4–4.0) in comparison to radiotherapy only (Table 5) [41]. However, 9 out of 12 studies showed no significant association between chemotherapy and radiation toxicity. One study found a significant association between chemotherapy and edema; however, no significant association between chemotherapy and pain or fibrosis (Table 6, Supplementary table B) [39]. Endocrine therapy without chemotherapy increased the risk of edema with 1.8 (95%CI 1.1–2.9) (Table 6) [41].

### 3.3. Patient Characteristics Associated with Late Radiation Toxicity

In one study, lower age was associated with general radiation toxicity and breast pain (Table 4) [19]. In another study, higher age was significantly associated with breast fibrosis (Table 5) [23]. The other 7 out of 10 studies investigating the association between late radiation toxicity and age found no significant association (Table 3) [21, 26, 29, 32, 34, 35, 39].

Larger breast size was associated with an increased risk of late radiation toxicity. A strong association between breast size > C or breast ptosis grade 2 or 3, resulting in a larger breast or larger footprint of the breast, and edema was reported (OR 5.34 95% CI 1.2–24.12), as well as a significant association between 1 L increase in breast volume and edema (OR 3.65 95% CI 2.54–5.24) (Table 6) [22]. Also, a larger breast size was independently associated with more toxicity in two studies, though different cut-off values were used: breast size >1500 cm^3^ (OR 2.10 95% CI 1.03–4.30) and breast size >1032 cm^3^ (OR 1.01 95% CI 1.00–1.03) [32, 34]. No association between breast size and late radiation toxicity was seen in 7 out of 13 studies (Supplementary table B–E). Also, the results for the association between tumor location, body mass index (BMI), and diabetes mellitus with late radiation toxicity were contradictory in several studies (Supplementary table C).

### 3.4. Other Factors Associated with Late Radiation Toxicity

A significant association between grade 1 general radiation toxicity and tobacco smoking was reported in one study (OR 2.15 95% CI 1.38–3.34) (Table 4) [37]. The same study also found a significant association between 3DCRT in comparison with accelerated hypofractionated radiotherapy technique (reported as MARA-1) and grade 1 and grade 2 general radiation toxicity, respectively (OR 2.18 95% CI 1.50–3.18 and 3.01 95% CI 1.08–8.42). One retrospective study found a favorable association between increasing tumor grade and fibrosis (OR grade 2 vs. 1 0.54 (95% CI 0.29–0.99); grade 3 vs. 1 0.29 (95% CI 0.11–0.74)) (Table 5) [24].

## 4. Discussion

The purpose of this systematic review was to provide an overview of factors associated with late radiation-induced breast toxicity after post-operative whole breast external beam irradiation in the modern 3D radiotherapy era. It is important to take factors associated with late radiation toxicity into account in order to evaluate new radiotherapy techniques. To our knowledge, no previous overview or systematic review was published on this topic. A higher radiotherapy dose or increased radiotherapy volume was associated with more late local radiation toxicity, as well as additional radiotherapy boost on the tumor bed. There was a wide variation in the way individual factors were defined and in the results of the studies included in this review. Due to heterogeneity of the data, high-quality evidence for factors associated with late radiation toxicity in breast cancer patients is therefore still lacking.

However, inconsistency between studies and study results made interpretation for this review difficult. The definition and measurement of determinants differed per study. For example, increased radiotherapy volume was defined as follows: increased radiotherapy volume measured (continuous), increase of volume per 10 cm^3^, increase PTV or CTV volume in different studies. Although we could conclude that increased radiotherapy dose or irradiated volume resulted in more toxicity, it was therefore difficult to draw other conclusions, such as a definite volume parameter, from these results. Also, the given breast cancer treatment varied per study. In some studies, all patients received a boost, whereas in other studies no boost was given. Furthermore, in most studies, patients were treated with breast conserving surgery, and in some studies, part of the study population was treated with mastectomy. Finally, there was a lot of variation in the study results. Especially in the category of patient characteristics, factors—such as age—could be significantly associated with late radiation toxicity in one study and not significant in another study. The heterogeneity of the results might be caused by several factors. First, different grading systems for late radiation toxicity were used in the included studies (e.g., RTOG criteria, CTCAE criteria, and LENT-SOMA scale). As the selected outcome method varies between the studies, the determinants associated with the outcome may also vary between studies. Second, the selection criteria of some cohort studies varied. For example the study of Bergom et al. only included patients with large breasts, the study of Meattini et al. only included patients <60 years old, while the study of La Rocca et al. only included patients >65 years old [24, 26, 33]. Consequently, the conclusions on patient characteristics might vary per study, as the accrued patient population also varied.

The methodology of the included studies caused some limitations. The way studies handled missing data was not reported in the majority of the studies. If no imputation method was used and missing cases were omitted in the analysis, there is a risk of selection bias, which could influence the outcome; therefore, all these studies scored a high risk of bias. Their results should be interpreted with caution. Also, there were 7/21 studies with a retrospective design, leading to a risk of bias. Patients with comorbidities or postoperative complications might have more extensive follow-up or patient files than patients without comorbidities or complications. As a consequence, their reports on late radiation toxicity could also be different; for example, in the study of Meattini et al. where a higher tumor grade was associated with less toxicity [24]. However, breast cancer patients with a grade 3 tumor receive no additional boost, in contrast to patients with grade 1-2. Potentially, toxicity was not caused by lower-tumor grade, but due to the absence of tumor bed boost, as adjustment for tumor boost was not performed.

Radiotherapy treatment has evolved greatly over the past decade. Hypofractionated radiotherapy has become the standard treatment in many countries, as different studies showed that it is a safe treatment option without increased toxicity in comparison to standard fractionation [10, 45, 46]. For example, in the START A trial, 2236 breast cancer patients were randomized to receive 41.6 Gy (13 fractions), 39.0 Gy (13 fractions), or 50 Gy (25 fractions, control group) [47]. After a median follow-up of 5 years, there was a trend toward less-patient reported toxicity (i.e., breast shrinkage, breast hardness, and swelling of the affected breast) in the groups receiving 41.6 Gy and 39.0 Gy in comparison to 50 Gy. However, no significant association between patient reported toxicity and radiotherapy was seen. Significantly less physician-reported breast induration and breast edema was seen in the group receiving 39 Gy in comparison to 50 Gy at 10 year follow-up [10]. In the START B trial, 2215 breast cancer patients were randomly assigned to receive 50 Gy in 25 fractions (control) or 40 Gy in 15 fractions (intervention) [45]. Again, a (nonsignificant) trend toward less-patient reported toxicity was seen in the group receiving a lower radiotherapy dose. However, at 10 years, follow-up significantly less breast shrinkage and breast edema was seen in the group receiving 40 Gy in comparison to the group receiving 50 Gy [10]. As a result, hypofractionated radiotherapy is implemented and part of standard care in the Netherlands. Simultaneously, new radiotherapy techniques, such as ultra-hypofractionation (i.e., five fractions) are developed, resulting in similar or lower toxicity rates [11, 48]. Also, partial breast irradiation is an upcoming treatment modality for patients with low-risk breast cancer. In the randomized IMPORT LOW study, partial breast radiotherapy resulted in significant less adverse events (incidence rate ratio 0.77), such as breast appearance, in comparison to 40 Gy whole-breast radiotherapy [49]. Also, patient reported breast appearance 5 years after radiotherapy was significantly better in the partial breast irradiation group (HR .064 95%CI 0.46–0.89) and reduced radiotherapy dose group (HR 0.74 95% CI 0.54–1.00) in contrast to whole breast group irradiated with 40 Gy [50]. In the Florence trial, patients were randomized to receive accelerated partial breast irradiation with IMRT or whole breast irradiation with 2D-RT [51]. The cosmetic outcome was significantly better in the partial irradiated group in contrast to whole breast irradiation (p 0.045). Also, less late radiation toxicity (any grade, using RTOG criteria) was reported in the partial breast group (p 0.004). However, as these trials are randomized trials, no patient- or treatment-related factors associated with late radiation toxicity were evaluated, and they were not included in our systematic review.

In the modern treatment area, like in 2D radiotherapy, increased radiotherapy dose and volume are associated with late radiation toxicity. We need to further explore if treatment adaptation and early intervention can prevent late radiation toxicity and knowing the factors that might induce late radiation toxicity, the possibility of individual treatment adaptation could be investigated and the effect of early intervention to prevent or reduce the risk of late radiation toxicity could be evaluated. Also, the optimal treatment for late radiation toxicity in breast cancer patients needs to be investigated.

## 5. Conclusion

Increased radiotherapy dose, including boost, or increased radiotherapy volume is associated with more late radiation toxicity after whole breast irradiation in the modern treatment era. It is important to further reduce late radiation toxicity rates without compromising locoregional control and survival, using treatment de-escalation such as partial breast irradiation patients receive a smaller total radiotherapy dose and selected use of tumor bed boost.

## Figures and Tables

**Table 1:** Study characteristics.

Study (year)	Patients (*n*)	FU duration (months)^a^	Age (years)^a^	Toxicity assessment	Study design	
Barnett (2011)	1014	24 (for all patients)	NR	RTOG criteria	RCT	Prospective
Bergom (2012)	109	40.3 (mean 45.9, range 1–127)	61 (27–91)	CTCAE 3.0	Cohort	Prospective
Bronsart (2017)	832	76.8 (18–148.8)	61.5 (29–90)	CTCAE 3.0	Cohort	Prospective
Ciamella (2014)	212	34 (8–44)	63 (39–88)^b^	RTOG criteria	Cohort	Prospective
De rose (2016)	144	37 (24–55)	62 (30–88)	CTCAE 4.0	Cohort	Prospective
De rose (2020)	831	2 years (at least)	60 (27–88)	RTOG and CTCAE	Cohort	Unclear
De santis (2016)	537	32	74 (46–91)	RTOG criteria	Cohort	Prospective
Digesu (2018)	447	52 (3–115)	63 (IQR 56–71)	RTOG criteria	Cohort	Retrospective
Hannan (2012)	129	9.87 (mean)	NP	RTOG criteria	Cohort	Retrospective
Hille-betz (2016)	159	19.4 (11.3–44.8)	58 (36–86)	LENT-SOMA	Cohort	Retrospective
Hosni (2017)	67	25 (11-34)	49 (31–69)	RTOG criteria	Cohort	Prospective
Ishiyama (2006)	193	45.6 (8–132)	50 (27–77)^b^	LENT-SOMA	Cross-sectional	Prospective
Joseph (2020)	175	73.1 (4.2–101.8)	58 (41–77); 59 (41–82)^c^	RTOG criteria	RCT	Prospective
Kelemen (2012)	198	28.8 (14.4–70.8)	62 (25–89)^b^	4-Point Likert	Cohort	Retrospective
Keller (2012)	946	31 (1–97)	58 (31–91)	Unclear	Cohort	Prospective
Lazzari (2017)	215	72	68 (60–75)^b^	RTOG criteria	Cohort	Retrospective
Lilla (2007)	421	51 (36–77)	61–70 (31–91)^d^	RTOG criteria and LENT-SOMA	Cohort	Prospective
Meattini (2019)	786	45.6 (24–102)	50 (22–60)	RTOG criteria	Cohort	Retrospective
Palumbo (2019)	220	12	62 (34–88)	CTCAE 4.03	Cohort	Prospective
La rocca (2019)	794	48.3 (6–114)	74 (65–91)	RTOG criteria	Cohort	Prospective
Yu (2017)	143	21.4 (3.8–61.6)	65 (44–91)	CTCAE 4.3	Cohort	Retrospective

^a^Numbers are shown as median (range), unless stated otherwise. ^b^Mean age.^c^In, respectively, inversed planned and helical tomography groups. ^d^Absolute number not provided, median extracted from data provided. Abbreviations: 3DCRT 3D conformal radiotherapy; CTCAE common terminology criteria for adverse events; IMRT intensity modulated radiotherapy; ILD isocentric lateral decubitus position; IQR inter-quartile range; LENT-SOMA late effects in normal tissues–subjective, objective, management and analytic score; RTC randomized controlled trial; RTOG radiation therapy oncology group; RTP radiotherapy; SIB simultaneous integrated boost; VMAT volumetric modulated arc radiotherapy; WBI whole breast irradiation.

**Table 2:** Overview of type of radiotherapy techniques and dose fractionation schedule in the included studies.

Study (year)	RTP technique	Prescribed RT dose	Boost	Nodal irradiation
Barnett (2011)	3DCRT	40 Gy in 15 fractions	Some patients	Some patients
Bergom (2012)	3DCRT prone position	45–50 Gy, fractionation unclear.	72% of patients (average 10 Gy in 5 fractions)	Unclear
Bronsart (2017)^a^	3DCRT (in lateral isocentric decubitus position)	47% 50 Gy + boost 18% 50 Gy in 25 fractions 26% 40–42.6 Gy in 13–15 fractions 10% 30 Gy in 5 fractions	47% 16 Gy sequential boost in 33 fractions	Unclear
Ciamella (2014)	3DCRT	40.05 Gy in 15 fractions.	26% of patients received sequential boost of 9 Gy in 3 fractions.	Unclear
De rose (2016)	VMAT	40.5 Gy	48.0 Gy concomitant boost in 15 fractions.	None
De rose (2020)	VMAT	40.5 Gy	48 Gy sequential integrated boost, 2.7 of 3.2 Gy/fraction	Unclear
De santis (2016)	3DCRT	42.4 Gy in 16 fractions	73% of patients receiving additional sequential boost (10 Gy in 4 fractions boost or 16 Gy in 8 fractions).	None
Digesu (2018)^b^	3DCRT vs. forward planning IMRT	50.4 Gy in 28 fractions 40 Gy in 16 fractions	Sequential boost 10 Gy in 4 fractions Concomitant boost 4 Gy	None
Hannan (2012)	Inverse planning IMRT	42.4 Gy in 16 fractions	9.6 Gy sequential boost in 4 fractions	None
Hille-betz (2016)^a^	3DCRT	57% 50 Gy in 25 fractions 43% 50.4 Gy in 28 fractions	32% of the patients received sequential boost	Unclear
Hosni (2017)	3DCRT	40 Gy in 15 fractions	Concomitant 3 Gy boost in 3 fractions	Unclear
Ishiyama (2006)	3DCRT	50 Gy in 25 fractions	Depending on protocol 10–16 Gy boost	Unclear
Joseph (2020)	Helical tomography IMRT vs. inverse planning IMRT	50 Gy in 25 fractions	None	None
Kelemen (2012)	3DCRT	50 Gy in 25 fractions	Some patients	Some patients
Keller (2012)^b^	Inverse planning IMRT	Median 46 Gy, fractionation unknown	99% of patients received concomitant boost (dose unknown)	Some patients
Lazzari (2017)	3DCRT	42.56 Gy in 16 fractions	None	Unclear
Lilla (2007)^b^	3DCRT	50 Gy in 25 fractions 50.4 Gy in 28 fractions 56 Gy with 2 Gy per fraction	5–20 Gy sequential boost	Unclear
Meattini (2019)^a^	3DCRT	43% 40 Gy in 15 fractions 57% 50 Gy in 25 fractions	Sequential 9–18.69 Gy boost in 3–7 fractions (some patients) Sequential 10–20 Gy boost in 5–10 fractions (some patients)	Unclear
Palumbo (2019)	3DCRT	42.4 Gy in 16 fractions	Sequential boost 10.6–13.25 Gy in 4-5 fractions (some patients)	
La rocca (2019)	3DCRT	42.4 Gy in 16 fractions	25% received sequential boost with 10–16 Gy in 4–8 fractions	Unclear
Yu (2017)	3DCRT	42.5 Gy in 16 fractions	8 Gy in 3 fractions	Unclear

Whole breast irradiation in all studies. If not reported in the table, dose, or fractionation was unknown. ^a^Not all patients received same radiotherapy dose. Proportion of patients receiving certain dose shown in third column.^b^Different radiotherapy doses administered, proportion of patients receiving certain dose unclear. Abbreviations: 3DCRT 3D conformal radiotherapy; IMRT intensity modulated radiotherapy; RT radiotherapy; SIB simultaneous integrated boost; VMAT volume modulated arc therapy; WBI whole breast irradiation.

**Table 3:** Overview of different domains of late radiation toxicity in relation to the summarized risk factors.

	Study	Radiotherapy		Surgery and systemic treatment					Patient characteristics							Other^a^
		Increased dose or irradiated volume	RT boost (dose)	Acute toxicity	RT- surgery interval	Surgical complications	ALND	Endocrine therapy	Chemotx	Other LRT symptoms	Age	Breast volume	Tumor location	BMI	Diabetes mellitus	
GRT	Ciamella	S/NS^b^	S/NS^b^	—	—	NS	—	—	S/NS^b^	—	NS	NS	—	—	NS	NS
	de rose	S	—	—	—	—	—	—	—	—	—	—	—	—	—	—
	de rose (2020)	NS	S	—	—	—	—	NS	NS	—	NS	S	—	—	—	S
	Digesu	S	—	—	—	—	—	NS	NS	—	—	—	—	—	NS/S^c^	S
	Hannan	S	—	—	—	—	—	—	—	—	—	S	—	S	—	S
	Hosni	—	—	—	—	—	—	—	—	—	S	NS	—	—	S	—
	Keller	S	S	—	—	—	—	S	S	—	—	—	—	—	—	—
	Lazzari	S	—	—	—	—	—	—	NS	—	—	NS	—	—	—	S
	Palumbo	—	NS	—	—	—	NS	NS	NS	—	—	—	—	—	NS	NS
	Yu	—	NS	—	—	S	—	—	—	—	NS	—	—	NS	—	S/NS
Pain	Barnett	—	S	S	—	S	—	—	—	—	S	—	—	—	—	—
	de rose (2020)	—	NS	—	—	—	—	—	—	—	—	NS	—	—	—	NS
	Hille-betz	NS	—	—	—	—	—	—	—	S	—	—	—	—	—	—
	Ishiyama	—	S	—	NS	—	—	—	NS	—	NS	—	NS	—	—	NS
Breast fibrosis	Bergom	—	NS	—	—	—	NS	—	NS	—	—	NS	—	NS	—	NS
	Bronsart	S/NS	—	—	—	—	—	—	NS	—	NS	NS	—	—	—	—
	de santis	NS	NS	—	—	—	—	—	NS	—	—	NS	—	—	NS	—
	Hille-betz	NS	—	—	S	—	—	—	NS	—	—	S	—	—	—	—
	Ishiyama	—	NS	—	S	—	—	—	NS	—	NS	—	NS	—	—	NS
	Joseph	—	—	—	—	—	—	NS	NS	NS	NS	S	—	—	—	NS
	Kelemen	S	—	—	—	—	S	—	—	S	—	—	—	—	—	S
	Lilla	—	—	—	—	—	—	—	—	—	S	—	—	—	—	S
	Meattini	NS	S	—	—	—	—	—	—	—	—	S	—	—	—	S/NS
	La rocca	—	S	—	—	—	—	—	NS	—	NS	—	—	—	—	—
Breast or arm edema	Barnett	—	S	S	—	—	—	—	—	—	S	S	—	—	—	—
	Hille-betz	NS	—	—	NS	—	S	—	—	—	—	S	—	NS	—	NS
	Ishiyama	—	NS	—	NS	—	—	—	S	—	NS	—	S	—	—	NS
	Kelemen	S	—	—	—	—	S	—	—	—	—	—	—	—	—	—
	Keller	S	S	—	—	—	—	S	S	—	—	—	—	—	—	—
	Meattini	S	S/NS^c^	—	—	—	—	—	—	—	—	NS	—	—	—	NS
	La rocca	—	S	—	—	—	—	—	—	—	NS	—	—	—	—	NS

^a^Depending on boost/no boost group. ^b^Depending on subacute or skin toxicity respectively. ^c^Depending on administered dose; Abbreviations: BMI body mass index; Chemotx chemotherapy; GRT late radiation toxicity; LRT late radiation toxicity; NS no significant association, RT radiotherapy; S significant association; Association not studied.

**Table 4:** Significant association between various risk factors and late radiation breast toxicity ≥12 months after whole breast irradiation.

Author (year)	Associated risk factors	Measure of association	Estimation of association
Ciamella (2014)	Skin toxicity	OR	
	Boost		3.06 (1.28–7.30)
	Subcutaneous toxicity		
	Chemotherapy		2.59 (1.17–5.72)
	Breast volumes receiving >104% vs. <100%		0.08 (0.1–0.52)
	Breast volumes receiving >107% vs.<100%		6.27 (1.34–29.37)
de rose (2016)	PTV	NR	
de rose (2020)	Boost volume > 70 cm^3^	OR	2.14 (1.26–3.62)
	Treated skin area^a^ > 400 cm^2^		2.16 (1.12–4.19)
	Breast size >1500 cm^3^		2.10 (1.03–4.30)
Digesu (2018)	Skin	OR	
	Tobacco smoking		2.15 (1.38–3.34)^b^
	PTV volume		1.12 (1.07–1.18)^b^
			1.27 (1.15–1.41)^c^
	Subcutaneous		
	3DCRT vs. Mara-1^d^ technique		2.18 (1.50–3.18)^b^
	Diabetes		3.01 (1.08–8.42)^c^
	PTV volume		1.65 (1.01–2.71)^b^
			1.14 (1.08–1.20)^b^
			1.14 (1.01–1.28)^c^
Hannan (2012)	Prone vs. supine position	R	NR
	Large breast vs small breast		NR
	BMI		0.38
	PTV		0.027
Hosni (2017)	Age >50 y vs. <50 y	OR	NR (1.01–1.20)
	DM^d^		NR (0.00–0.20)
Keller (2012)	RT boost dose (>16 vs. <16 Gy)	OR	2.4 (1.5–3.7)
	RT vs. RT combined with chemotherapy and endocrine therapy		1.9 (1.2–2.9)
	CTV 500–900 vs. <500		1.9 (NR)
	CTV ≥900 vs. <500		3.0 (2.0–4.5)
	Boost energy ≥12 MeV vs. ≤10		1.8 (1.3–2.7)
Lazzari (2017)	PTV <1300 vs. >1300 cc	R	0.955
	Breast volumes receiving >110%^f^		0.402
	Surgery good vs. poor result		0.455
Palumbo (2018)	None	HR	NA
Yu (2017)^g^	Re-excision	NR	
	Postoperative complication		

All shown variables were significantly associated with late radiation toxicity. See Supplementary material for nonsignificant variables. ^a^Skin surface surrounding irradiated area receiving at least 20 Gy. ^b^Estimation for Grade 1 toxicity. ^c^Estimation for Grade 2 toxicity. ^d^Modulated accelerated hypofractionated radiotherapy. ^e^No vs. yes^f^<10% vs. >10%. ^g^Results of univariable analysis, no multivariable analysis performed. Abbreviations: 3DCRT 3D conformal radiotherapy; BMI body mass index; CTV clinical target volume; DM diabetes mellitus; MeV megaelectrovolt; NA not applicable; NR not reported; OR odds ratio; PTV planned target volume; R Pearson's correlation; RT radiotherapy.

**Table 5:** Significant association between different risk factors and breast fibrosis in irradiated breast cancer patients ≥12 months after whole breast irradiation.

Author (year)	Associated risk factors	Measure of association	Strength of association
Bergom (2012)	None	NA	
Bronsart (2017)	Radiotherapy dose 50 Gy vs. 30 Gy	OR	12.5 (2.73–57.13)
De santis (2016)	None	NA	
Hille-betz (2016)	Ptosis grade 2/3 or C-cupsize	NR	0.02^a^
	Interval to radiotherapy		0.03^a^
Ishiyama (2006)^b^	Time after surgery (<2 vs. >5 years)	OR	0.06 (0.005–0.83)
Kelemen (2012)	100 cm^3^ increase irradiated breast volume	OR	1.07 (1.00–1.14)
	10 cm^3^ increase boost volume		1.12 (1.09–1.33)
	Photon boost	NR	
	Edema	NR	
	PTV	NR	
Joseph (2020)	Breast volume (<1032 cm^3^ vs. >1032 cm^3^)	OR	1.01 (1.00–1.03)
Lilla (2007)	Age		1.06 (1.01–1.11)
	Allergy		2.45 (1.11–5.51)
Meattini (2019)	Extensive intraductal component	OR	2.15 (1.17–3.98)
	Tumor grade 2 vs. 1		0.54 (0.29–0.99)
	Tumor grade 3 vs. 1		0.29 (0.11–0.74)
	Breast size >492 cc		2.64 (1.50–4.65)
	Boost dose >10 Gy		6.76 (2.04–22.45)
La rocca (2019)	Boost	OR	1.70 (1.16–2.48)

All shown variables were significantly associated with late radiation toxicity. See Supplementary material for nonsignificant variables. ^a^*p*-value ^b^Reported outcome is breast firmness. Abbreviations: NA not applicable; NR not reported; OR odds ratio; PTV planned target volume.

**Table 6:** Significant association between different risk factors and edema in irradiated breast cancer patients ≥12 months after breast cancer treatment.

Author (year)	Associated risk factors	Measure of association	Strength of association
Barnett (2011)	Breast volume (1 L increase)	OR	3.65 (2.54–5.24)
	Age		1.44 (1.18–1.76)
	Boost		1.71 (1.20–2.43)
	Acute toxicitya		1.51 (1.13–2.02)
		
		
		
Hille-Betz (2016)	Arm edema	OR	
	Axillary lymph node dissection		4.3 (1.4–13.58)
	Breast edema		
	Axillary lymph node dissection		10.59 (2.1–53.36)
	Ptosis grade 2/3 or bra size>C		5.34 (1.2–24.12)
Ishiyama (2006)^b^	Chemotherapy	OR	5.64 (1.18–26.98)
	Supraclavicular RTc		16.03 (3.06–84.01)
	Parasternal RTc		13.92 (2.16–89.90)
Kelemen (2012)	10 cm3 increase boost volume	OR	1.21 (1.09–1.33)
	Tumor size	NR	
	Axillary lymph node dissection	NR	
	Fibrosis	NR	
	Asymmetry	NR	
Keller (2012)	Boost dose (>16 vs.<16 gy)	OR	1.9 (1.2–3.0)
	Boost energy >12 MeV vs. <10 MeV		1.8 (1.3–2.7)
	RTP alone vs. RTP, chemotherapy, and endocrine therapy		2.3 (1.4–4.0)
	RTP alone vs. RTP and endocrine therapy		1.8 (1.1–2.9)
	CTV <500 vs. 500–900 cc		2.1 (1.4–3.2)
	CTV <500 vs. >900 cc		4.7 (2.9–7.5)
Meattini (2019)	Hypofractination	OR	0.18 (0.04–0.75)
	Boost dose >10 Gy		15.43 (2.08–114.3)
La Rocca (2019)	Boost	OR	1.70 (1.08–2.67)

All shown variables were significantly associated with late radiation toxicity. See Supplementary material for nonsignificant variables. ^a^Per unit increase in RTOG score measured at week 3. ^b^Reported outcome is thickening of arm. ^c^No vs. yes. Abbreviations: CTV clinical target volume; L liters; NR not reported; OR odds ratio; RT radiotherapy.

**Table 7:** Significant association between different risk factors and breast pain in irradiated breast cancer patients ≥12 months after breast cancer treatment.

Author (year)	Associated risk factors	Measure of association	Estimation of association
Barnett (2011)	Breast pain	OR	
	Boost		1.38 (1.04–1.83)
	Age		0.81 (0.70–0.94)
	Oversensitivity		
	Postoperative infection		1.78 (1.27–2.49)
	Acute toxicity		1.29 (1.02–1.64)
De rose (2020)	None	NA	
Hille-betz (2016)	Lymphedema arm^b^	OR	3.9 (1.17–13.5)
Ishiyama (2006)	Boost	OR	3.30 (1.26–8.66)

All shown variables were significantly associated with late radiation toxicity. See Supplementary material for nonsignificant variables. ^a^Shoulder/arm pain Abbreviation: OR odds ratio.

## Data Availability

Data are available upon reasonable request.

## Supplementary Materials

Supplementary Materials Supplementary material (A). Search string. Supplementary table (B). Association between pain and nonsignificant variables per study. Supplementary table (C). Association between general radiation toxicity and nonsignificant variables per study. Supplementary table (D). Association between fibrosis and nonsignificant variables per study. Supplementary table (E). Association between edema and nonsignificant variables per study.
