# Supplementary material for: Factors Associated with Late Local Radiation Toxicity after Post-Operative Breast Irradiation
Source: Breast J. 2022 Apr 16;2022:6745954. doi: 10.1155/2022/6745954 (PMC9187272; doi:10.1155/2022/6745954)
Supplement: Supplementary Materials — Supplementary material (A). Search string. Supplementary table (B). Association between pain and nonsignificant variables per study. Supplementary table (C). Association between general radiation toxicity and nonsignificant variables per study. Supplementary table (D). Association between fibrosis and nonsignificant variables per study. Supplementary table (E). Association between edema and nonsignificant variables per study. [file 6745954.f1.docx]

**Supplementary material**

**Supplementary material A**. Search string

**Supplementary table B**. Association between pain and non-significant variables per study

**Supplementary table C**. Association between general radiation toxicity and non-significant variables per study

**Supplementary table D**. Association between fibrosis and non-significant variables per study

**Supplementary table E**. Association between edema and non-significant variables per study

**Supplementary material A.** Search string

Search string Pubmed

((((((((Late[Title/Abstract]) OR (long-term[Title/Abstract]) OR (longterm[Title/Abstract])) AND (((Radiotherapy[MeSH Major Topic])OR(Radiotherap*[Title/Abstract]) OR (radiation treatment[Title/Abstract]) OR (Radiation[MeSH Major Topic]) OR (Radiation[Title/Abstract]) OR (Irradiation*[Title/Abstract]) OR (Radiation-induced[Title/Abstract]) OR (Radio-induced[Title/Abstract]) OR (irradiated[Title/Abstract])) AND ((Tissue injury[Title/Abstract]) OR (tissue injuries[Title/Abstract]) OR (Toxicity[Title/Abstract]) OR (Toxicities[Title/Abstract]) OR (CTCAE[Title/Abstract]) OR (Damage[Title/Abstract]) OR (Necrosis[Title/Abstract]) OR (Oedema[Title/Abstract]) OR (Edema[Title/Abstract]) OR (Lymphedema[Title/Abstract]) OR (lymphoedema[Title/Abstract]) OR (Fibrosis[Title/Abstract]) OR (Complication*[Title/Abstract]) OR (side effect*[Title/Abstract]) OR (adverse effect*[Title/Abstract]) OR (adverse event*[Title/Abstract]) OR (delayed adverse effect [Title/Abstract]) OR (delayed toxicity [Title/Abstract])))) AND ((Breast Neoplasms[MeSH Major Topic]) OR (breast neoplasm*[Title/Abstract]) OR (breast cancer*[Title/Abstract]) OR (mammary cancer*[Title/Abstract]) OR (mamma cancer*[Title/Abstract]) OR (breast tumor*[Title/Abstract]) OR (breast tumour*[Title/Abstract]) OR (mammary tumour*[Title/Abstract]) OR (mammary tumor*[Title/Abstract]) OR (mamma tumor*[Title/Abstract]) OR (mamma tumour*[Title/Abstract]) OR (mammary neoplasm*[Title/Abstract]) OR (mamma neoplasm*[Title/Abstract]) OR (breast carcinoma*[Title/Abstract]) OR (mammary carcinoma*[Title/Abstract]) OR (mamma carcinoma*[Title/Abstract]) OR (breastcancer*[Title/Abstract]) OR (Breast malignan*[Title/Abstract])))))))

Search string Embase

('breast cancer'/exp OR 'breast neoplasm*':ti,ab OR 'breast cancer*':ti,ab OR 'mammary cancer*':ti,ab OR 'mamma cancer*':ti,ab OR 'breast tumor*':ti,ab OR 'breast tumour*':ti,ab OR 'mammary tumour*':ti,ab OR 'mammary tumor*':ti,ab OR 'mamma tumor*':ti,ab OR 'mamma tumour*':ti,ab OR 'mammary neoplasm*':ti,ab OR 'mamma neoplasm*':ti,ab OR 'breast carcinoma*':ti,ab OR 'mammary carcinoma*':ti,ab OR 'mamma carcinoma*':ti,ab OR 'breastcancer*':ti,ab OR 'breast malignan*':ti,ab) AND ('late':ti,ab OR 'long-term':ti,ab OR 'longterm':ti,ab) AND ('radiation toxicity'/exp OR 'skin toxicity'/exp OR 'tissue injury':ti,ab OR 'tissue injuries':ti,ab OR 'toxicity':ti,ab OR 'toxicities':ti,ab OR 'ctcae':ti,ab OR 'damage':ti,ab OR 'necrosis':ti,ab OR 'oedema':ti,ab OR 'edema':ti,ab OR 'lymphedema':ti,ab OR 'lymphoedema':ti,ab OR 'fibrosis':ti,ab OR 'complication*':ti,ab OR 'side effect*':ti,ab OR 'adverse effect*':ti,ab OR 'adverse event*':ti,ab OR ‘delayed adverse effect’:ti,ab OR ‘delayed toxicity’:ti,ab) AND ('radiotherapy'/exp OR 'radiotherap*':ti,ab OR 'radiation treatment':ti,ab OR 'radiation'/exp OR 'radiation':ti,ab OR 'irradiation*':ti,ab OR 'radiation-induced':ti,ab OR 'radio-induced':ti,ab OR 'irradiated':ti,ab) AND [embase]/lim NOT [medline]/lim

**Supplementary table B.** Association between pain and non-significant variables per study

| **Author (year)** | **Associated risk factors** | **Measure of association** | **Estimation of association** |
| --- | --- | --- | --- |
| Barnett (2011) | NA | NA |  |
| De Rose (2020) | Breast >1000cm3  TSA >400cm  Boost volume >70cm3 | NR |  |
| Hille-Betz (2016) | Radiation dose including boost | OR | 1.08 (0.997-1.164) |
| Ishiyama (2006) | Age <46 vs. 46-60  Type of surgery  Time after surgery <2 vs. 2-5 vs >5 years  Chemotherapy  Supraclavicular RT  IMN RT  T-stage 0-1 vs. 2-4 | OR | NR |

Abbreviations: IMN Internal mammary lymph nodes; NA not applicable (i.e. all variables were significant); NR not reported; OR odds ratio; RT radiotherapy; TSA treated skin area.

**Supplementary table C.** Association between general radiation toxicity and non-significant variables per study

| **Author (year)** | **Associated risk factors** | **Measure of association** | **Estimation of association** |
| --- | --- | --- | --- |
| Ciamella (2014) | Skin toxicity  Chemotherapy  Hypertension  Age  Breast volume  Diabetes  Surgical deficits  Breast volumes receiving >100% vs. <100%  Breast volumes receiving  >104% vs. <100%  Breast volumes receiving >107% vs. <100%  Boost volumes receiving  >100% vs. <100%  Boost volumes receiving  >104% vs. <100%  Boost volumes receiving  >107% vs. <100%  Subcutaneous toxicity  Hypertension  Age  Breast volume  Diabetes  Surgical deficits  Boost administration  Breast volumes receiving >100% vs. <100%  Boost volumes receiving >100% vs.<100%  Boost volumes receiving >104% vs.<100%  Boost volumes receiving >107% vs.<100% | NR^a^ | 0.232  0.898  0.087  0.692  0.139  0.890  0.642  0.466  0.908  0.981  0.684  0.615  0.731  0.223  0.483  0.055  0.499  0.298  0.745  0.728  0.099  0.585 |
| de Rose (2016) | NA | NA |  |
| de Rose (2020) | Volume >5cm3 receiving >105%  Chemotherapy  Hormonal therapy  Age  Breast volume receiving >105% | NR |  |
| Digesu (2018) | Skin  MARA 1 technique  Hypertension  Diabetes  Alcohol  Chemotherapy  Hormone therapy  Subcutaneous  Hypertension  Tobacco smoking  Alcohol  Chemotherapy  Hormone therapy | NR |  |
| Hannan (2012) | NA | NA |  |
| Hosni (2017) | Breast volume | NR |  |
| Keller (2012) | NA | NA |  |
| Lazzari (2017) | Breast diameter  Chemotherapy | R | 0.110  -0.085 |
| Palumbo (2018) | Hypofractionated boost  Nodes excised >10  Chemotherapy | HR | 1.05 (0.65-1.69)  1.49 (0.91-2.45)  1.46 (0.88-2.42) |
| Yu (2017) | Age  BMI  Oncoplastic closure  Excision volume  Re-excision  Boost technique  Boost dose | NR^a^ | 0.14  0.57  0.99  0.10  0.07  0.14  0.76 |

^a^ No measurement of association provided, p-value reported.

Abbreviations: BMI body mass index; HR hazard ratio; NA not applicable (i.e. only significant results); NR not reported.

**Supplementary table D.** Association between fibrosis and non-significant variables per study

| **Author (year)** | **Associated risk factors** | **Measure of association** | **Strength of association** |
| --- | --- | --- | --- |
| Bergom (2012) | BMI  Caucasian vs. African American race  Chemotherapy  Lymph nodes dissected none vs. 1-5 vs. >6  Lymph nodes dissected none vs >1  Boost  Excisional volume  Breast volume | NR^a^ | 0.70  0.21  0.11  1.0  1.0  0.18  0.17  0.66 |
| Bronsart (2017) | Age at diagnosis  Chemotherapy  Cup size | OR | NR |
| de Santis (2016) | Diabetes  Chemotherapy  Breast volume <0.553 vs. 0.553-806.9 vs. >806.9  Breast volume receiving >median value  Boost | OR | 1.2 (NR)  1.4 (NR)  1.2 (NR)  2.1 (NR)  1.5 (NR) |
| Hille-Betz (2016) | PTV  Chemotherapy | NR | 0.058  0.091 |
| Ishiyama (2006)^a^ | Age <46 vs. 46-60  Type of surgery  Boost  Chemotherapy  Supraclavicular RT  IMN RT  T-stage 0-1 vs. 2-4 | OR | NR |
| Joseph (2020) | Age (<66 vs >67 years)  Inversed planned IMRT vs HT IMRT  Moist desquamation  Dmax (<55Gy vs >55Gy)  Tumor stage  Lymphovascular space invasion  Hormonal treatment  Adjuvant chemotherapy | OR | 0.97 (0.94-1.01)  1.07 (0.53-2.19)  1.27 (0.46-3.52)  6.66 (0.78-56.77)  NR  NR  NR  NR |
| Kelemen (2012) | NA | NA |  |
| Lilla (2007) | NA | NA |  |
| Meattini (2019) | Hypofractionation  Ki67 index<20% vs. >20%  Breast volume receiving >107% vs. <107% | OR | 0.88 (0.35-2.22)  1.23 (0.61-2.51)  1.00 (0.44-2.27) |
| La Rocca (2019) | Age 70-74 vs. 65-69  Age 75-79 vs. 65-69  Age >80 vs. 65-69  CCI 1 vs. 0  CCI >2 vs. 0 | OR | 0.63 (0.40-1.01)  0.69 (0.42-1.14)  0.78 (0.45-1.35)  1.10 (0.69-1.74)  1.54 (0.98-2.41) |

^a^ Patient reported breast firmness

Abbreviations: IMN Internal mammary lymph nodes; NA not applicable; NR not reported (i.e. only p-value reported); OR odds ratio

**Supplementary table E.** Association between edema and non-significant variables per study

| **Author (year)** | **Associated risk factors** | **Measure of association** | **Strength of association** |
| --- | --- | --- | --- |
| Barnett (2011) | NA | NA |  |
| Hille-Betz (2016) | Arm edema  BMI  Resection volume  PTV  Breast edema  PTV  Interval between surgery and RTP | NR | 0.07  0.06  0.13  0.10  0.09 |
| Ishiyama (2006)^a^ | Age <46 vs. 46-60  Type of surgery  Time after surgery <2 vs. 2-5 vs. >5 years  Boost  T-stage 0-1 vs. 2-4 | NR | NR |
| Kelemen (2012) | NA | NA |  |
| Keller (2012) | NA | NA |  |
| Meattini (2019) | EIC presence vs. absence  Boost dose <10Gy vs. none  Breast size >492cc vs. <492cc  Breast volume receiving >107% vs. <107% | OR | 1.06 (0.54-2.12)  0.98 (0.12-8.11)  1.70 (0.80-3.58)  1.84 (0.53-6.37) |
| La Rocca (2019) | Age 70-74 vs. 65-69  Age 75-79 vs. 65-69  Age >80 vs. 65-69  CCI 1 vs. 0  CCI >2 vs. 0 | OR | 0.65 (0.37-1.17)  0.94 (0.53-1.69)  0.87 (0.44-1.69)  0.88 (0.51-1.52)  1.16 (0.68-1.97) |

^a^ patient reported thickening of arm

Abbreviations: BMI body mass index; CCI charlson comorbidity index; EIC extensive intraductal component; NA not applicable; NR not reported (i.e. when provided only p-values reported); OR odds ratio; PTV planned target volume; RTP radiotherapy
